# Assessment of Knowledge and Attitudes Toward HPV and HPV Vaccine Among Students of a University in Cyprus: A Cross-Sectional Survey

**DOI:** 10.3390/vaccines14100885

**Published:** 2026-10-08

**Authors:** Burcu Ozbakir, Duru Dulger, Lara Sawah

**Affiliations:** 1 Department of Obstetrics and Gynaecology, Faculty of Medicine, Near East University, Nicosia 99138, Cyprus; 2 DESAM Research Institute, Near East University, Nicosia 99138, Cyprus; 3 Biological Sciences, Faculty of Life Sciences, University College London, London WC1E 6BT, UK; duru.dulger07@gmail.com; 4 Faculty of Medicine, Near East University, Nicosia 99138, Cyprus; laramhdsawah@gmail.com

**Keywords:** HPV vaccine, HPV, university students, survey, Cyprus

## Abstract

**Background/Objectives:** This study aims to determine university students’ knowledge and attitudes in Cyprus regarding human papillomavirus (HPV) and the HPV vaccine. **Methods:** We conducted a cross-sectional study of 601 university students in Cyprus between March 2025 and June 2025 using quota-based convenience sampling. Undergraduate students in the faculties of Medicine, Nursing, Midwifery, and Engineering completed a detailed questionnaire assessing HPV and HPV vaccine knowledge and attitudes, as well as variables including gender, age, year of study, field of study, and self-reported racial/ethnic categories as operationalised in the survey, age at first sexual intercourse, sexual orientation, smoking, and history of number of sexual partners. The questionnaire assessed knowledge about HPV and the HPV vaccine, as well as attitudes and the self-reported reasons behind them. **Results:** Females and health-care students showed higher awareness of HPV and the HPV vaccine. Knowledge also increased with higher year of study and age. Among the variables examined, students aged over 24 had the highest vaccination rate. The most cited self-reported reasons for receiving the vaccine were to prevent cervical cancer and genital warts. Attitudes towards the HPV vaccine varied markedly across variables. **Conclusions:** HPV knowledge is directly related to preventive efforts, and young adults are key actors. Further research into attitudes towards the HPV vaccine and the reasons behind them may increase vaccination and decrease HPV-related cancers globally.

## 1. Introduction

Human papillomavirus (HPV) is the most common sexually transmitted pathogen, and the infection is responsible for many types of cancers among all genders [1]. Although almost all sexually active and unvaccinated people are predicted to be infected with HPV, 90% of them will develop natural immunity [2]. In the rest, the infection will persist and may cause anogenital warts and specific HPV-related cancer types (cervical, vaginal, vulvar, penile, oropharyngeal, and anal cancers) [3]. Among these, the most common is cervical cancer in women and oropharyngeal cancer in men. Cervical cancer, the fourth most frequent cancer among females, is the most common cause of cancer-related deaths in developing countries [4]. That is why general HPV knowledge in the population gains importance.

HPV is transmitted via oral, vaginal, or anal contact through skin-to-skin contact, mostly during sexual intercourse. The asymptomatic nature of the primary infection and the ease of transmission increase the risk of infection [5]. The factors associated with HPV infection risk are lifetime sexual partner number, sexual partner number before the age of 21, first sexual intercourse at a younger age, history of another sexually transmitted disease, younger age, hormonal contraception use, and smoking, which indicates that HPV knowledge of young adults is essential for controlling HPV transmission [6].

Currently, HPV prevention is achievable through immunisation. In 2026, several HPV vaccines are available [5]. The World Health Organization (WHO) advises HPV vaccination for all genders between the ages of 9–26, as almost half of all new infections are seen between the ages of 15–24 [7]. WHO primarily targets girls aged 9–14, preferably before sexual intercourse initiation [2]. The case for boys and older populations varies between national policies, vaccination programmes, vaccine availability, and resources. The Centres for Disease Control and Prevention also recommended Gardasil 9 for use between the ages of 29–45 for both genders in the US [8].

The HPV vaccination target for 2030 is 90% of girls fully vaccinated by age 15 as the WHO cervical cancer elimination strategy; however, in 2023 in the European region, the percentage was still 30%, though improving [9]. For adolescents, parents generally decide about vaccination, but university students can decide for themselves [4]. The hesitancy of parents is partially due to the fear of their children being involved in sexual intercourse earlier and the anti-vaccine movement. For university students, hesitancy mainly stems from limited knowledge, underestimating personal risk, costs, and conservative socio-cultural values [7]. International university students have an additional risk with language barriers, less social integration, and lack of family support, leading to increased high-risk sexual behaviours and less medical/educational support [4,7].

Awareness and attitudes towards HPV infection and the vaccine are assessed across the general population, but literature specifically focusing on young adults is limited [10,11]. Furthermore, to the authors’ knowledge, published evidence from Cyprus remains limited. This study is unique in surveying university students in Cyprus and evaluates demographic information, HPV knowledge, and attitudes towards the HPV vaccine. By exploring these underlying factors causing HPV vaccination hesitancy, strategic programmes on HPV education and promoting HPV vaccination may be developed.

In Cyprus, HPV vaccines are licenced, and the south offers a free, publicly funded HPV vaccine and vaccination programme for youth, while the north had no consistent, free, publicly funded national immunisation programme for HPV until 2025, although there have been pilot studies and earlier attempts [12,13,14]. The university evaluated in the study is in the north, where free publicly funded HPV vaccine and vaccination started only in 2025; and is still not free of charge for males [13]. These participants were ineligible in north Cyprus for the free vaccination because of their age. The HPV vaccine and vaccination are recommended in the immunisation programme in north Cyprus for adults aged 26 and below, although not free of charge [15]. Research conducted in Cyprus found that total HPV vaccination coverage was 38.1% among female university students [14].

## 2. Materials and Methods

### 2.1. Participants

**Figure 1:**
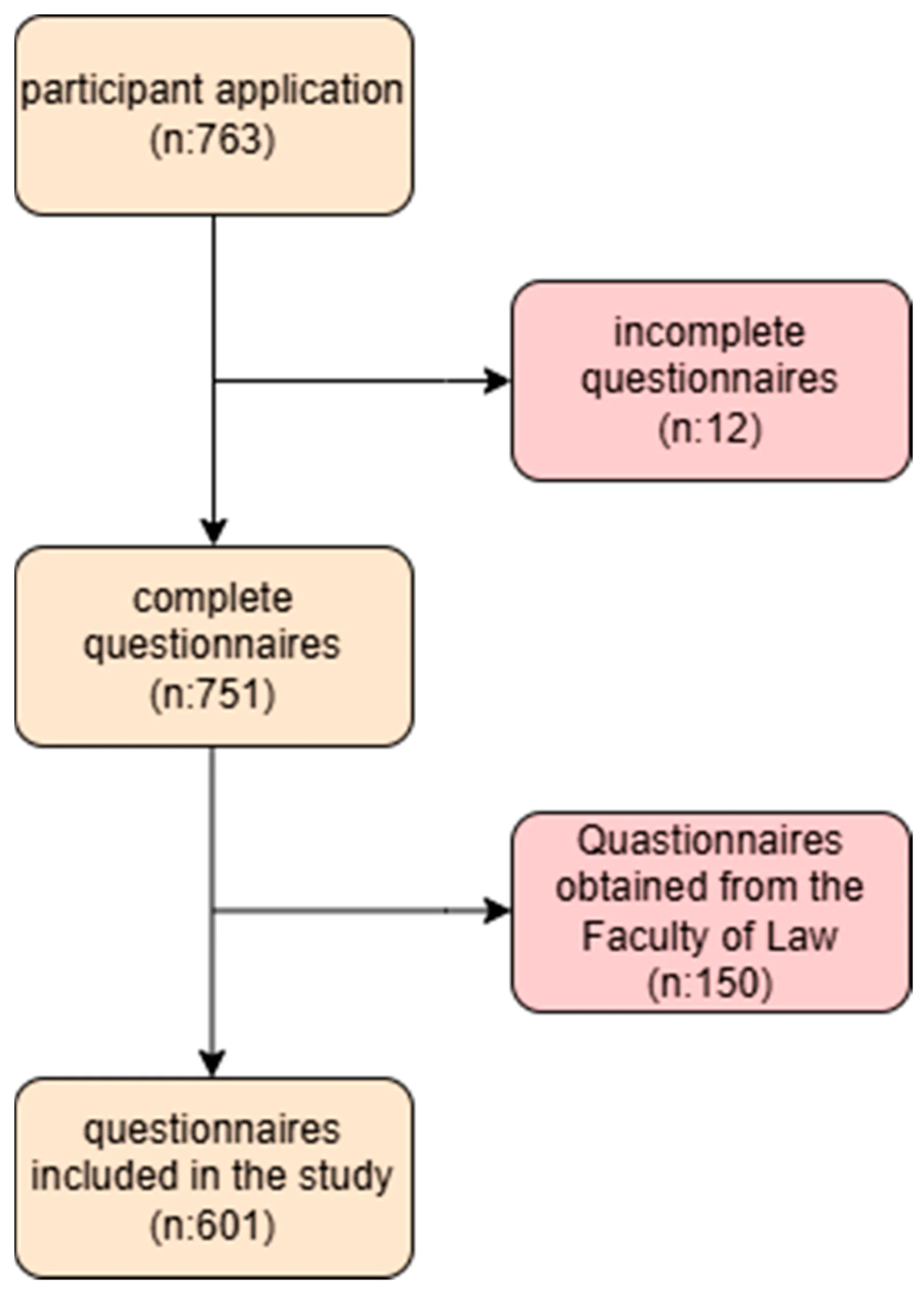
Flowchart of the participant selection.

The study population comprised undergraduate students at Near East University in Cyprus. We recruited the participants using quota-based convenience sampling between March 2025 and June 2025 on weekdays and within school hours. A female volunteer offered translated and back-translated Turkish and English questionnaires to all students of the Medicine, Nursing, Midwifery, Law, and Engineering faculties at the faculty canteens. We set up stands in the canteens of Medicine, Nursing, Midwifery, Law, and Engineering, in that order. We set up stands at the faculty canteens, and 763 students from the selected faculties approached the stands. At the end of each day, the volunteer checked if all the questionnaires were fully completed and added them to the list of accepted responses. Incomplete questionnaires (*n* = 12) were not considered. When each faculty reached 150–151 completed questionnaires, questionnaire collection stopped. We collected similar numbers of participants from each faculty to ensure an equal distribution of participants across educational departments. The inclusion criterion was being an undergraduate student in one of the chosen faculties. Exclusion criteria included incomplete questionnaires and student applications from faculties without research approval. The Faculty of Law was excluded because, although it had initial ethical approval, the relevant dean did not approve using questionnaires from law students for this study (Figure 1).

### 2.2. Data Collection

The study used a cross-sectional design. Participants completed a 22-question survey designed for this study. The questions were informed by other surveys on this topic and were reviewed by a gynaecology expert, although they were not copied directly [7,8,10,11]. The questionnaire collected demographic and personal information, basic knowledge of HPV and its vaccine, HPV vaccination, and attitudes towards the HPV vaccine (Supplementary Materials S1–S3). Because our survey questions were not taken from a validated questionnaire, we could assess only general knowledge, awareness of the consequences, attitudes toward the HPV vaccine, and the self-reported reasons for vaccine hesitancy using our own questionnaire data.

Knowledge and awareness were defined based on HPV word knowledge, knowing that it is a common sexually transmitted disease and that it does not show clinical signs in most cases. Also, knowledge of HPV-caused disease, how HPV is transmitted, and what the HPV vaccine can prevent were assessed.

We defined attitude as motivations for HPV vaccination, self-reported hesitancy or reasons for non-vaccination, and recommendations of the HPV vaccine to others, such as a relative, a friend, a child, or an adolescent.

Questions were multiple-choice or required numerical input. We piloted the questionnaire with 10 medicine students before the main questionnaire; we included them in the final analysis because we did not change the questionnaire after the pilot test. Volunteers completed the questionnaire anonymously in a separate area of the canteen and returned it.

We did not use a scoring system since we created the questionnaire ourselves. For multiple-choice questions, we considered each choice separately and evaluated the percentage of correct answers for each choice. We did not treat the “I do not know” response as incorrect; instead, we evaluated it as a separate category alongside correct and incorrect answers. The item-level analyses were exploratory.

During the 2024–2025 academic year, student registration across Near East University’s Faculties of Medicine, Nursing, Midwifery, and Engineering totalled 2780 students [16]. The minimum sample size required to detect an effect in a population of 2780, with a 95% confidence interval and a 5% margin of error, is 338 [17]. The total response rate of the four faculties was 613 out of 2780 (22.1%).

### 2.3. Ethics

The Near East University Medical Faculty Ethical Committee approved the study for the faculties of Medicine, Nursing, Midwifery, Law, and Engineering (Supplementary Materials S4). With ethical approval, we collected questionnaires from all faculties. However, after collection, the dean of the Law Department refused to allow use of the questionnaire for unknown reasons. We subsequently shredded the questionnaires collected from law students and excluded them from analysis.

### 2.4. Statistical Analysis

We analysed research data using IBM SPSS Statistics Version 27.0 [18]. We summarised categorical variables using counts and percentages. We assessed the normality of numerical variables using skewness and kurtosis, and considered values within the ±2 range acceptable for a normal distribution [19].

We used the Pearson chi-square test to assess relationships between categorical variables. If any expected cell frequency was less than 5, we used Fisher’s exact test. For multiple-response questions, we treated each response option as a separate category and compared response distributions. We presented categorical results as counts (%). In significant chi-square analyses, we evaluated effect sizes using Cramér’s V. Because Fisher’s exact test relies on precise probability calculations, we did not report a conventional test statistic in these analyses. Furthermore, we did not calculate Cramér’s V for comparisons using Fisher’s exact test; we reported only exact *p*-values.

The primary outcomes were awareness of HPV, awareness of the HPV vaccine, vaccination, and recommending the vaccine to children/adolescents or friends/relatives. We considered other subgroup comparisons based on demographic characteristics as exploratory.

In cross-tabulation analyses, we used binary logistic regression to evaluate the independent effects of independent variables that showed a significant relationship with the dependent variable. We included variables significant at *p* < 0.05 in Pearson chi-square or Fisher’s exact tests in the relevant logistic regression model. We created a separate model for each binary outcome variable. In multivariate binary logistic regression models, variables were selected not based on *p*-values in cross-tables, but considering the research question, their scientific and epidemiological significance, and potential confounding factors. Age and gender were included in all models because they represent basic demographic characteristics; the year of entry presents the students’ position in the educational process, and the field of education represents differences in exposure to HPV-related educational content. Ethnicity was included in the HPV awareness, HPV vaccine awareness, and vaccine recommendation models to control for potential demographic differences. Since the HPV vaccination model included 69 events, adding ethnicity would increase the number of parameters in the model and reduce the event/parameter ratio from approximately 9.9 to 6.9. Therefore, we did not include ethnicity in this model. Six participants who reported their gender as “Other” were excluded from the regression models due to the small size of this group. Six participants who reported their ethnicity as Hispanic/Latin were combined with the “Other” ethnicity category because the number was insufficient for a separate coefficient estimation. The combined category consisted of 44 participants, and no separate impact estimate was made for the Hispanic/Latin group.

No imputation was applied to the “age at first sexual intercourse” variable, and participant responses were not subsequently altered based on assumptions. In descriptive and comparative analyses, we evaluated each variable based on its number of valid responses. For the age at first sexual intercourse variable, 247 participants had responses available, while 354 participants had missing information. For the lifetime number of sexual partners variable, no data were missing (N = 601). In total, 359 participants reported 0, 165 participants reported 1–5, 34 participants reported 6–10, and 43 participants reported more than 10 lifetime sexual partners. Data control revealed that five participants who reported a lifetime number of sexual partners as 0 also reported an age at first sexual intercourse. Because these two pieces of information came from separate self-report questions and no independent verification source existed to determine which response was incorrect, we made no corrections to these five participants’ responses, and we did not remove them from the dataset. Both variables were analysed using their own reported values and their own valid denominators. Since age at first sexual intercourse and lifetime number of sexual partners were not included in the multivariate logistic regression models, this discordance in responses did not affect the analytical sample size of the regression models.

Categorical independent variables were included in the model using appropriate reference categories. We presented results as odds ratios (OR), 95% confidence intervals, and *p*-values. We evaluated model fit with the Hosmer–Lemeshow test and reported Nagelkerke R^2^ values. We set the statistical significance level at 0.05.

## 3. Results

### 3.1. Sample Characteristics

A total of 601 students completed the questionnaire. Most participants were female, followed by males and a few other genders. There was no age limit, but participants were grouped into three categories, with the most common being 20–24. Most participants were in the middle of their education. Education fields included medicine, nursing, midwifery, and engineering, with equal participants from each. Self-reported racial/ethnic categories as operationalised in the survey were White ethnicity, Black or African ethnicity, Hispanic or Latino ethnicity, Asian ethnicity, and others, with White self-reported ethnicity having the most participants. Age at first sexual intercourse was recorded numerically, then categorised as <18 years and ≥18 years, with >18 slightly more common. Sexual orientation was grouped as heterosexual or LGBTIQ, with mostly heterosexual participants. Smoking status was assessed as current smokers, former smokers, and never smokers, with the most common group being never smokers. Total sexual partner count varied from zero to more than ten, with zero being the most common choice. Interestingly, the number of participants with a history of sexual intercourse was 247, but only 242 answered more than 0 to the number of sexual partners question, so 5 participants reported 0 sexual partners despite stating they had sexual activity. The analysis of age at first sexual intercourse is based on the 247 respondents who provided valid responses. The numerous subgroup analyses in Table 1 are exploratory (Table 1).

### 3.2. HPV Knowledge Parameters

We evaluated whether the participants had heard of HPV before, and 78.5% replied positively (Table 2). Among the variables, gender, age, year and field of education, self-reported racial/ethnic categories as operationalised in the survey, and age at first sexual intercourse were statistically significant regarding HPV knowledge. Awareness was higher among those aged >20 years. Awareness was higher among students in their senior and middle years, medical, nursing, and midwifery students (health-care students), participants of White self-reported ethnicity, and participants who had their first sexual intercourse at age ≥18 (Supplementary Materials S5).

Regarding knowledge of HPV frequency, female participants and health-care students knew more. As participants’ age and educational attainment increased, their knowledge increased. Participants of White self-reported ethnicity knew more than those of other self-reported ethnicities. Participants with >10 sexual partners also had better knowledge (Supplementary Materials S5).

When we carried out multivariable logistic regression analysis, HPV awareness was found to be significantly predicted by gender, years of education, and total number of sexual partners (*p* < 0.05). When female participants were considered as a reference, male participants had a lower probability of HPV awareness (OR = 0.199, *p* < 0.001). The “other” gender category also showed a lower probability of HPV awareness compared to women (OR = 0.073, *p* = 0.006) (Supplementary Materials S6).

Compared to first-year students, middle-year students had a 2.65 times higher probability of HPV awareness (OR = 2.650, *p* = 0.001). In senior students, this probability was 4.23 times higher (OR = 4.230, *p* < 0.001). Participants reporting 1–5 total sexual partners also had a higher probability of HPV awareness (OR = 2.094, *p* = 0.009). No significant association was found between sexual orientation, smoking, 6–10 partners, and 11 or more partners categories and HPV awareness (Supplementary Materials S6).

Women, health-care students, and participants of White self-reported ethnicity showed increased awareness regarding the asymptomatic tendency of HPV. Again, as the educational year and age increased, knowledge increased (Supplementary Materials S5).

Women, participants of White self-reported ethnicity, and health-care students had greater knowledge of HPV-related medical conditions. Regardless of age, higher education was associated with greater knowledge (Supplementary Materials S5). Female participants, health-care students, and participants of White and Asian self-reported ethnicity had better HPV transmission-route knowledge than the other participants.

### 3.3. HPV Vaccine Knowledge Parameters

We evaluated whether the participants had heard of HPV vaccine before, and 69.9% replied positively (Table 2). Among the variables, gender, year and field of education, self-reported racial/ethnic categories as operationalised in the survey, and total sexual partner count were statistically significant for HPV vaccine awareness. Female participants and increasing years of education were positively associated with awareness. Health-care students, participants of White self-reported ethnicity, and participants with fewer than six sexual partners were also more aware (Supplementary Materials S7).

In the multivariate logistic regression analysis of HPV awareness, after controlling for age, gender, years of education, field of study, and ethnicity, HPV awareness was associated with field of study and ethnicity. Compared to engineering students, the rate of having heard of HPV was significantly higher in medical, nursing, and midwifery students (*p* < 0.001). HPV awareness was negatively correlated with Black or African ethnicity and Asian participants compared to White ethnicity participants. We found no significant association in the “Other” ethnicity group (*p* > 0.05). We found no significant association between age, gender, years of education, and HPV awareness (*p* > 0.05). The disappearance of the gender difference observed in the univariate analysis when field of study was controlled for suggests that this difference largely stems from the predominantly female health sciences programmes (Supplementary Materials S6).

We present the results of a multivariate logistic regression analysis of HPV vaccine awareness. After controlling for other variables, HPV vaccine awareness was associated with gender, field of education, and ethnicity. Awareness of the HPV vaccine was lower in men than in women. HPV vaccine awareness was positively associated with medical, nursing, and midwifery students compared to engineering students (*p* < 0.05). It was negatively associated with Black or African ethnicity participants and the “Other” ethnicity group compared to White ethnicity participants (*p* < 0.05). No significant association was found in Asian participants (*p* = 0.924). No significant association was found between age, years of education, and HPV vaccine awareness (*p* > 0.05). The association observed in the previous model for years of education did not remain significant when field of education and age were controlled for together (Supplementary Materials S8).

### 3.4. HPV Vaccine Attitude Parameters

We evaluated whether the participants had received the HPV vaccine before, and 11.6% self-reported having received it (Table 2). Among the variables, only participants’ age was associated with HPV vaccination history; those aged over 24 had the highest vaccination rate. In multivariable logistic regression, although the model as a whole was significant, its explanatory power was low. After controlling for other variables, vaccination status was associated with age, gender, and years of education (*p* < 0.05). Each 1-year increase in age was positively associated with vaccination rate (*p* < 0.05). Vaccination rates were lower in men than in women (*p* < 0.05). Vaccination rates were lower in middle-year students than in junior students, and we found no significant correlation for final-year students (*p* > 0.05). We found no significant correlation between field of education and vaccination status (*p* > 0.05) (Supplementary Materials S9).

We evaluated recommendations for HPV vaccine for a child/adolescent, and 47.6% responded positively. We also evaluated recommendations for the HPV vaccine to a relative/friend, and 64.2% responded positively (Table 2). Recommending the HPV vaccine to a child/adolescent, a relative/friend was higher among women, senior students, health-care students, and participants of White self-reported ethnicity. Participants with increasing age and first sexual experience at or over eighteen were more likely to advise the vaccine to a relative/friend (Supplementary Materials S7).

The results of a multivariate logistic regression analysis regarding the likelihood of recommending the HPV vaccine to children or adolescents are presented. After controlling for other variables, educational background and ethnicity were associated with the likelihood of recommending the vaccine. Medical and midwifery students had higher odds of recommending the HPV vaccine compared to engineering students. The relationship was similar among nursing students but was not statistically significant (*p* > 0.05). Black participants had lower odds of recommending the vaccine compared to white participants (*p* < 0.05). No significant association was found in Asian (*p* = 0.741) and “Other” (*p* = 0.413) ethnicity groups. Age, gender, and years of education were not significantly associated with the likelihood of recommending the vaccine (*p* > 0.05). The differences observed in univariate analysis by gender and years of education did not remain significant after controlling for educational background (Supplementary Materials S10).

The multivariable regression results for the likelihood of recommending the HPV vaccine to a friend or relative are presented. After controlling for other variables, the likelihood of recommendation was associated with age, gender, and field of education. Each 1-year increase in age was positively associated with the recommendation rate (*p* < 0.05). The recommendation rate was lower in men than in women (*p* < 0.05). The recommendation rate was significantly higher among medical, nursing, and midwifery students than among engineering students (*p* < 0.001). No significant correlation was found between years of education (*p* > 0.05), ethnicity, and the likelihood of recommendation (*p* > 0.05). The lower odds observed in Black or African ethnicity participants did not reach statistical significance (*p* > 0.05) (Supplementary Materials S11).

The most well-known conditions preventable by the HPV vaccine were HPV-related cervical cancer and genital warts. Females, senior students, health-care students, and participants who self-reported White or Asian ethnicity demonstrated greater knowledge.

Participants’ motivations for HPV vaccination (routine vaccination at school, prevention of HPV-related cancers, prevention of transmission to the partner, prevention of genital warts, knowing someone with HPV, knowing someone with HPV-related cancer, and knowing someone who had HPV vaccination) were evaluated. None of the variables showed a significant relationship with the motivations (*p* > 0.05).

We evaluated participants’ motivations and self-reported reasons and barriers to HPV vaccination. Because non-vaccinated participants could select more than one self-reported reason for not getting the HPV vaccine, some percentages may exceed 100%.

**Figure 2:**
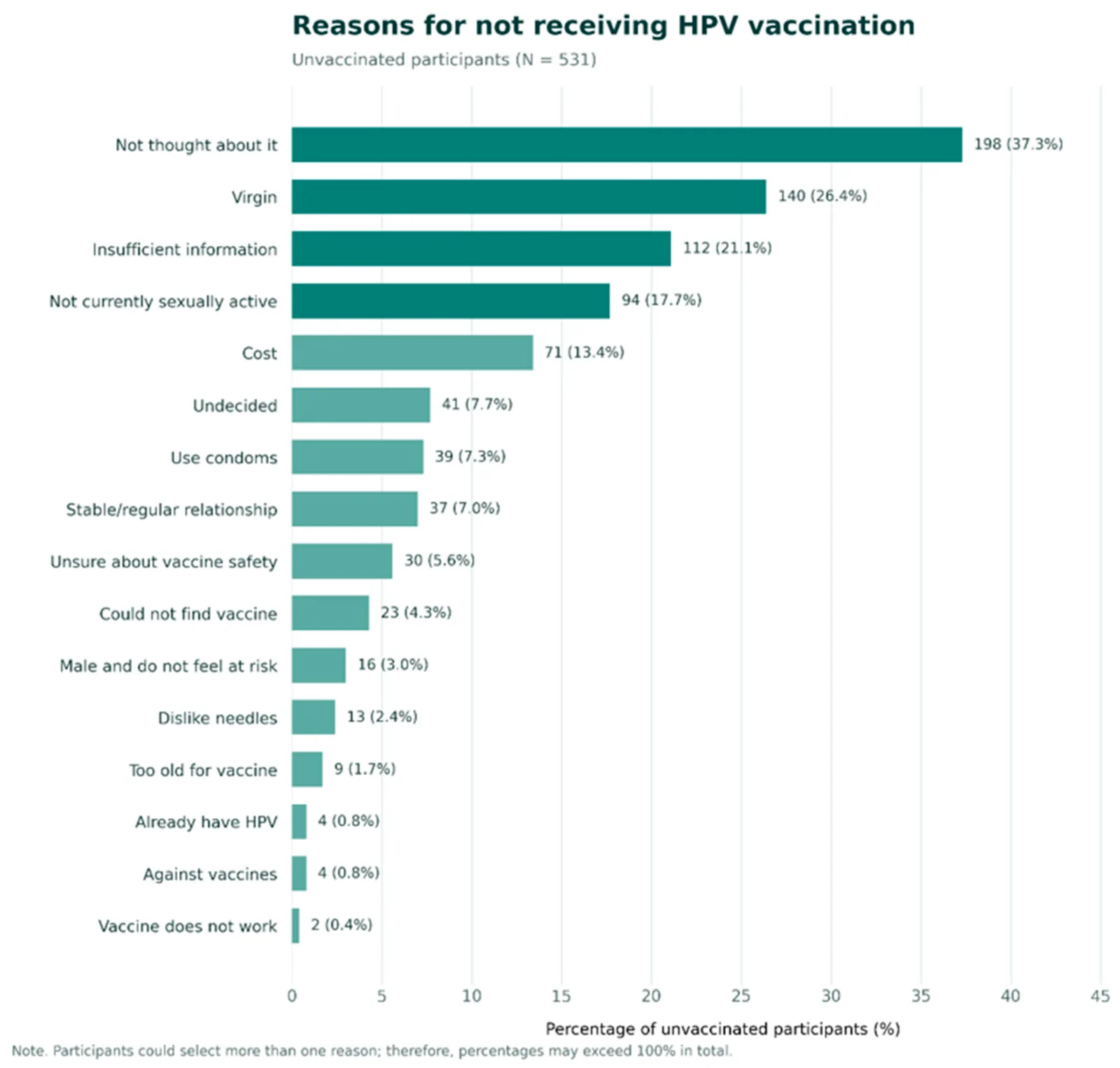
Reasons for not getting the HPV vaccine among unvaccinated participants (N = 531).

Among those who did not receive the HPV vaccine, all participants, regardless of gender, admitted they had not thought about it yet, which was the most frequent choice (Figure 2).

The relationships between demographic and sexual health characteristics of participants who did not receive the HPV vaccine and their multiple responses regarding reasons for not getting the vaccine were presented. Among 531 participants who did not receive the HPV vaccine, the most frequently reported reason was “I have never thought about this” (*n* = 198, 37.3%). This was followed by “I am a virgin” (*n* = 140, 26.4%), “I do not have enough information about the vaccine” (*n* = 112, 21.1%), and “I am not currently sexually active” (*n* = 94, 17.7%). A total of 71 participants cited the cost of the vaccine as a reason (13.4%). The distribution of responses differed descriptively across subgroups. While “I am a virgin” (31.7%) and “I am not currently sexually active” (23.3%) responses were higher in women, “I do not have enough information” (28.7%) and “I use condoms” (15.9%) responses were more prominent in men. Among participants under 20, the responses “I am a virgin” (34.7%) and lack of information (31.9%) were more prevalent, while among those aged 25 and over, the response “I use condoms” (15.9%) was more common. Regarding year of education, first-year students showed a higher percentage of “lack of information” (29.7%), middle-year students showed “I am a virgin” (31.4%), and final-year students showed “I have never thought about this” (42.2%). Regarding field of study, engineering students showed a higher percentage of “lack of information” (37.3%), while midwifery students showed higher percentages of “vaccine cost” (29.9%) and “not being sexually active” (27.6%). Regarding sexual orientation, LGBTIQ participants showed a higher percentage of “lack of information” (31.0%), “indecision” (20.7%), and “condom use” (17.2%). Current smokers showed higher percentages of “lack of information” (25.0%) and “condom use” (15.8%). Among those without a sexual partner, the response “I am a virgin” (37.7%) was more common, while among those with more than 10 partners, the responses “I have never thought about this” (52.6%) and “I use condoms” (34.2%) were more prevalent. Among 208 unvaccinated participants for whom information on age of first sexual intercourse was available, those who started having sexual intercourse before the age of 18 showed a higher percentage of responses such as “I have never thought about this” (43.2%), while those who started at age 18 and later showed higher percentages of responses such as regular/stable intercourse (17.5%) and cost (17.5%) (Supplementary Materials S12) (Figure 2).

### 3.5. HPV and HPV Vaccine Knowledge Sources

We examined sources of HPV and HPV vaccine knowledge. Overall, 43.9% of participants chose teachers as their information source, the most common source (Table 3). Among females, the top three were teachers, health-care personnel, and media. Among males, the top three were media, health-care personnel, and teachers. From juniors to seniors, the sources shifted from media and teachers to health-care personnel and teachers. Health-care students preferred health-care personnel and teachers, whereas non-health-care students preferred media and others. Students who self-reported White ethnicity preferred teachers and health-care personnel, whereas students who self-reported Black or African ethnicity preferred media and teachers (Supplementary Materials S13).

## 4. Discussion

Knowledge of HPV and its vaccine is especially important in young adults who can choose to take protective measures. Most studies evaluated public knowledge, but few have examined university students. Evaluating young adults may show the real debates about HPV vaccine and vaccination hesitancy [8,11,20].

One strength is its diverse sample of 601 students, reflecting a wide range of demographic and personal characteristics. To the authors’ knowledge, the studies comparing health-care and non-health-care fields of education, age at first sexual intercourse, sexual orientation, and number of sexual partners are limited. The study focused not only on basic HPV knowledge but also on how that knowledge translated into attitudes.

Like female participants, health-care students had better knowledge, and studies including only females or health-care students would yield one-sided results [21,22,23]. Male students also lacked knowledge of HPV risk in males. The study also showed that the male participants “do not feel at risk” more often. Therefore, HPV and HPV vaccine’s importance should not be focused only on cervical cancer in educational materials. Interestingly, sexual orientation did not affect HPV knowledge or attitudes, which was inconsistent with known data [10,24].

Some participants saw condom use as a complete preventive method. Although condoms cannot eliminate HPV transmission, they can reduce transmission [25]. That is why condoms cannot replace the HPV vaccine or cervical screening.

Participants knew about HPV at 78.5% and the HPV vaccine at 69.9%, but vaccination was only 11.6%, similar to other studies, showing that knowledge is not the only motivation for vaccination and that hesitancy needs reassessment [26,27].

In our study, having 1–5 sexual partners was positively associated with HPV knowledge, but having 6–10 sexual partners was negatively associated with HPV vaccine knowledge. The possible link between having fewer sexual partners and increased sensitivity to HPV should be studied further. The possibility that older age at first sexual intercourse could be associated with better knowledge is consistent with previous studies [22,28].

Both increasing age and year of education seemed to improve knowledge of HPV and its risks [8]. To what extent campus life could be the link to this, since it could be associated with self-learning, social interaction, and access to the health-care system, consistent with known data, should be studied further. Age was only related to increased vaccination, which may reflect access to the health-care system over time.

The study found a link between self-reported racial/ethnic categories, as operationalised in the survey, and HPV knowledge, with participants who self-reported White ethnicity having the highest knowledge and a better attitude toward the vaccine than those who self-reported other ethnicities. Further research should examine whether these differences across ethnic groups relate to limited access to health-care, language and educational barriers, affordability, and vaccination policies [28,29,30].

Regarding self-reported reasons and barriers for non-vaccination, midwifery students said the vaccine was expensive, medicine students said they preferred to avoid sexual intercourse, and non-health-care students reported a lack of knowledge about the vaccine. Similar studies show that HPV vaccine cost is an issue for females, which also applies to northern Cyprus, where the vaccine is not free for adults [13,31].

Advising a child or adolescent about the vaccine was less effective than advising a friend or relative, consistent with known data [20,32]. This shows that simply knowing about the vaccine is not enough, and that simple educational materials about early vaccination need to be provided. Interestingly, vaccination coverage remained at 11.6%, whereas the recommendation was between 47.6% and 64.2%. This may suggest barriers such as cost and the burden of accessing the vaccine as well.

Personal vaccination hesitations differed by gender, with male participants reporting less knowledge; the reason behind this should be studied further in northern Cyprus. With increasing age, condom use was replaced by abstaining from sex for some time; temporary abstinence and condom use do not replace vaccination or routine cervical cancer prevention strategies over the life course.

Sources of knowledge were mainly teachers, health-care professionals, and media. Males and participants of Black or African self-reported ethnicity chose media as their main learning source; the reliability and accuracy of the sources should be studied further [8,20]. As the education progressed, the source shifted from media to health-care personnel as well. The ethnicity-related variations are exploratory.

As a limitation, multiple comparisons in bivariate and multivariable logistic regression analyses increase the risk of type I error.

Another limitation is that we recruited all participants from a single university, which may not reflect the broader university population in Cyprus. Another limitation was that our sample included only participants who approached the stand; therefore, it may not represent the whole faculty and may introduce selection bias. Those who were not in the canteen or did not wish to participate in a study about this topic may come from different backgrounds and hold different knowledge, beliefs, or prejudices about HPV.

Another limitation was that we did not evaluate each participant’s overall knowledge score and assessed only the correct percentages for each answer choice. As a result, we may have missed a more accurate assessment of knowledge with a verified questionnaire, as our questionnaire categories were broad, heterogeneous, and not designed to identify causal explanations. Our questionnaire also did not directly measure language, country of prior residence, educational background, health literacy, health-care access, or vaccination policy.

The discrepancy between the number of participants declaring to have at least one sexual partner and the ones reporting their age at their first sexual activity may be attributed to a misunderstanding of the question wording, inconsistent self-reporting, or varying interpretations of the term “sexual partner” among participants. As the bivariate analysis of age at first sexual intercourse (N: 247) and sexual partner counts (N: 242 for more than 0) were insignificant, we did not include these parameters in the multivariable regression analysis.

Another limitation was that the study did not include the Middle Eastern ethnicity option, which is part of the university student community. Future studies regarding Cyprus should include Middle Eastern as well as more specific ethnic categories or provide an open-ended self-reported ethnicity option in the questionnaire.

Another limitation was that we did not verify participants’ self-reported vaccination status against medical records and accepted their self-reported answers as correct. Some participants may have misremembered their vaccination status. A social-desirability bias may also have played a part, despite the survey being anonymous. We also did not check for potential repeated participation by the same participant.

Another limitation was that we did not use a standard, pre-made HPV questionnaire; instead, we created a broader questionnaire from scratch for this study. Therefore, we could not obtain a score for our variables. Another limitation was that we did not ask about HPV-related disease history, which could have affected knowledge and attitudes. We also had small subgroup sizes to compare, and the questionnaire did not adjust for confounding factors.

As another limitation, we did not separate LGBTIQ participants according to sexual orientation and gender identity variations. Therefore, we may not have captured gender identity-variant participants correctly.

Future studies that include several universities, faculties, and socio-cultural backgrounds may yield more generalisable results.

## 5. Conclusions

In this cross-sectional survey of students at one university, awareness of HPV and the HPV vaccine varied by several demographic and educational characteristics, while self-reported HPV vaccination uptake was low. Because the study used quota-based convenience sampling, relied on self-reported information, and used a questionnaire developed for this study, interpret the findings as exploratory and do not generalise them to all university students in Cyprus. Future multicentre studies using validated multilingual instruments should investigate the roles of vaccine access, cost, health literacy, country of prior residence, national vaccination policy, and risk perception. Educational interventions should include all genders and be combined with strategies to improve access to HPV vaccine and vaccination. HPV education may encourage preventive actions, with the two most important groups to receive this education being parents and young adults. Focusing on these actors and understanding their knowledge and attitudes may help reduce HPV-related cancers globally.

## Figures and Tables

**Table 1:** Characteristics of the participants.

Variable	Group	*n*/N	%	Valid *n*	Missing *n*
Gender	Female	380/601	63.2	601	0
	Male	215/601	35.8		
	Other	6/601	1.0		
Age group	<20	82/601	13.6	601	0
	20–24	432/601	71.9		
	>24	87/601	14.5		
Year of education	Junior	87/601	14.5	601	0
	Middle	325/601	54.1		
	Senior	189/601	31.4		
Field of education	Medicine	150/601	25.0	601	0
	Nursing	150/601	25.0		
	Midwifery	151/601	25.1		
	Engineering	150/601	25.0		
Self-reported racial/ethnic categories as operationalized in the survey	White ethnicity	370/601	61.6	601	0
	Black or African ethnicity	159/601	26.5		
	Hispanic or Latino	6/601	1.0		
	Asian	28/601	4.7		
	Other	38/601	6.3		
Age at first sexual activity	<18	100/601	40.5	247	354
	≥18	147/601	59.5		
Sexual orientation	Heterosexual	567/601	94.3	601	0
	LGBTIQ	34/601	5.7		
Smoking	None	362/601	60.2	601	0
	Quit	66/601	11.0		
	Smoking	173/601	28.8		
Sexual partner number	0	359/601	59.7	601	0
	1–5	165/601	27.5		
	6–10	34/601	5.7		
	>10	43/601	7.2		

Values are presented as the number of observations (%). The total sample size is N = 601. Valid *n* represents the number of valid responses for the relevant variable; missing *n* represents the number of missing responses. Percentages were calculated based on the number of valid responses for each variable. For age at first sexual intercourse, there were 247 valid responses and 354 missing data points. There were no missing data points for other variables. Five participants who reported 0 lifetime sexual partners also reported age at first sexual intercourse. Since these two variables were separate self-report questions and there was no independent data to confirm which response was incorrect, the responses were retained unchanged, and each variable was analysed based on its own valid denominator.

**Table 2:** Total participants’ HPV awareness, awareness of HPV vaccine, self-reported vaccination status, and HPV vaccine recommendation attitudes.

Questions	Yes *n*/N	Yes %	95.5 CI
Have you heard of HPV?	472/601	78.5	75.1–81.7
Have you heard about the HPV vaccine?	425/601	70.7	66.9–74.4
Have you received an HPV vaccine?	70/601	11.6	9.2–14.6
Do you recommend the HPV vaccination for a child/adolescent?	286/601	47.6	43.5–51.7
Do you recommend the HPV vaccination to your relatives/friends?	386/601	64.2	60.2–68.1

CI: confidence interval. Percentages show the proportion of participants in the total sample who reported the relevant response category. Confidence intervals were calculated at 95%.

**Table 3:** Sources of information about HPV and HPV vaccine of all participants.

	Family	Friends	Health-Care	Teachers	Media	Other
	*n*/N (%)	*n*/N (%)	*n*/N (%)	*n*/N (%)	*n*/N (%)	*n*/N (%)
Total Participants	70/601 (11.6)	121/601 (20.1)	236/601 (39.3)	264/601 (43.9)	226/601 (37.6)	106/601 (17.6)

Respondents could select more than one answer, and the percentages may exceed 100%.

## Data Availability

The raw data supporting the conclusions of this article will be made available by the authors on request.

## Supplementary Materials

The following supporting information can be downloaded at https://www.mdpi.com/article/10.3390/vaccines14100885/s1. Supplementary Materials S1. HPV questionnaire (in English); Supplementary Materials S2. HPV questionnaire (in Turkish); Supplementary Materials S3. The correct answers of the questionnaire; Supplementary Materials S4. Scientific Research Ethics Committee Research Project Evaluation Report; Supplementary Materials S5. Associations between participant characteristics and HPV awareness, knowledge of HPV prevalence, and awareness of asymptomatic infection; Supplementary Materials S6. Multivariable logistic regression analysis of factors associated with HPV awareness; Supplementary Materials S7. Associations between participant characteristics, awareness of the HPV vaccine, self-reported vaccination status, and HPV vaccine recommendation attitudes; Supplementary Materials S8. Multivariable logistic regression analysis of factors associated with HPV vaccine awareness; Supplementary Materials S9. Multivariable logistic regression analysis of factors associated with HPV vaccination; Supplementary Materials S10. Multivariable logistic regression analysis of factors associated with recommending the HPV vaccine to a child/adolescent; Supplementary Materials S11. Multivariable logistic regression analysis of factors associated with recommending the HPV vaccine to a friend/relative; Supplementary Materials S12. Self-reported reasons for not receiving HPV vaccine among unvaccinated participants; Supplementary Materials S13. Sources of information about HPV and HPV vaccine among subgroups.

## Abbreviations

The following abbreviations are used in this manuscript:

HPV	Human papillomavirus
WHO	World Health Organization
IBM	International Business Machines Corporation
SPSS	Statistical Package for the Social Sciences
LGBTIQ	Lesbian, Gay, Bisexual, Transgender, Intersex, and Queer
