# Supplementary material for: Assessment of Knowledge and Attitudes Toward HPV and HPV Vaccine Among Students of a University in Cyprus: A Cross-Sectional Survey
Source: Vaccines (Basel). 2026 Oct 8;14(10):885. doi: 10.3390/vaccines14100885 (PMC13709563; doi:10.3390/vaccines14100885)
Supplement: Supplementary file 1 [file vaccines-14-00885-s001.zip › S1. HPV questionnaire (English).pdf]

Data Collection Form about HPV Knowledge among University Students in Cyprus

Dear Participant,
This study was designed to determine HPV knowledge among university students in Cyprus. Your answers will be kept confidential, and the research data will not be used for anything other than scientific purposes. Thank you for your participation in the study.

Near East University Faculty of Medicine
Responsible researcher: Asst.Prof. Burcu Özbakır, MD, MSc
Contact:
Tel: 05338880580
E-mail: drburcuburcu@hotmail.com
Points to consider when answering the questionnaire:
Answer each multiple-choice question by ticking a single choice that best reflects you or your knowledge. Just choose the option closest to you.
If the multiple-choice question includes the information “You can select all the choices you think are correct.”, you can tick as many choices as you wish.
For open-ended questions, write the number specified in the questions in the relevant area.

1. What is your gender?
A) Female
B) Male
C) Other

2. What is your age? ________

3. What is your year of education?
A) First year
B) Middle years
C) Last year

4. What is your major education?
A) Medicine
B) Nursing
C) Midwifery
D) Law
E) Engineer

5. What is your race?
A) White
B) Black or African
C) Hispanic or Latino
D) Asian
E) Other

6. Have you heard of HPV (Human papillomavirus) before?
A) Yes
B) No

7. Is HPV a very common sexually transmitted disease?
A) Yes
B) No
C) I do not know

8. Does an HPV infection always show clinical signs?
A) Yes
B) No
C) I do not know

9. Which one/ones of the below can be caused by HPV? (You can select all the choices you think are correct.)
A) I have no idea
B) Cervical cancer
C) Genital warts
D) Vaginal cancer
E) Vulvar cancer
F) Anal cancer
G) Penis cancer
H) Head and neck cancer
I) HIV or AIDS

10. How can HPV be transmitted? (You can select all the choices you think are correct.)
A) I have no idea
B) Holding hands
C) Kissing
D) Genital skin-to-skin contact
E) Public toilets
F) Unprotected vaginal sex
G) Unprotected oral sex
H) Unprotected anal sex
I) Sex with a condom
J) Sharing a spoon or cup
K) Sneezing or coughing

11. Have you heard of the HPV vaccine before?
A) Yes
B) No

12. Which of the one/ones below may be prevented with HPV vaccination? (You can select all the choices you think are correct.)
A) I have no idea
B) Cervical cancer
C) Genital warts
D) Vaginal cancer
E) Vulvar cancer
F) Anal cancer
G) Penis cancer
H) Head and neck cancer
I) HIV or AIDS

13. If you have heard of HPV and HPV vaccines, where did you learn about them? (You can select all the choices you think are correct.)
A) Family
B) Friends
C) Healthcare providers
D) Teachers
E) Media
F) Other

14. Have you had an HPV vaccination?
A) Yes
B) No

15. If you have had the vaccine (if you answered "yes" to the 14th question), what motivated you? (You can select all the choices you think are correct.)
A) Routine vaccination at school
B) To prevent cervical/other cancers
C) To prevent the spread of HPV to my partner
D) To prevent warts
E) I know someone who has or had HPV
F) I know someone who has or had cancer because of HPV
G) I know someone who has received the HPV vaccine
I) I do not know

16. If you haven't had the vaccine (if you answered "No" to the 14th question), why not? (You can select all the choices you think are correct.)
A) I have never really thought about it
B) I do not know enough about it
C) I am in a stable relationship
D) I am a virgin
E) I am not sexually active right now
F) I am too old for the vaccine
G) It is expensive
H) I am not sure it is safe
I) I could not find the vaccine
J) I am still undecided
K) I do not like needles
L) I am against vaccines
M) The vaccine does not work
N) I am a male and do not feel at risk
O) I already have HPV
P) I am using condoms

17. Would you recommend the HPV vaccine for a child or adolescent?
A) Yes
B) No
C) I do not know

18. Would you recommend the HPV vaccine to a friend or a relative?
A) Yes

B) No
C) I do not know

19. What is your lifetime sexual partner number?
A) 0
B) 1-5
C) 6-10
D) more than 10

20. Age the first time you had sex (if you had)? ________

21. What is your sexual orientation?
A) Heterosexual
B) LGBTIQ

22. What is your smoking history?
A) Never smoked
B) Quit smoking
C) Current smoker

Thank you for filling in the questionnaire :)
