# Supplementary material for: Assessment of Knowledge and Attitudes Toward HPV and HPV Vaccine Among Students of a University in Cyprus: A Cross-Sectional Survey
Source: Vaccines (Basel). 2026 Oct 8;14(10):885. doi: 10.3390/vaccines14100885 (PMC13709563; doi:10.3390/vaccines14100885)
Supplement: Supplementary file 1 [file vaccines-14-00885-s001.zip › S2. HPV questionnaire (Turkish).pdf]

Kıbrıs'taki Üniversite Öğrencileri Arasında HPV Bilgisi Hakkında Veri Toplama Formu

Değerli Katılımcı,
Bu çalışma Kıbrıs'taki üniversite öğrencileri arasında HPV bilgisini belirlemek için tasarlanmıştır. Cevaplarınız gizli tutulacak ve araştırma verileri bilimsel amaçlar dışında hiçbir amaçla kullanılmayacaktır. Çalışmaya katıldığınız için teşekkür ederiz.

Yakın Doğu Üniversitesi Tıp Fakültesi
Sorumlu araştırmacı: Yrd.Doç.Dr. Burcu Özbakır, MD, MSc
İletişim:
Tel: 05338880580
E-posta: drburcuburcu@hotmail.com
Anketi cevaplarken dikkat edilmesi gereken noktalar:
Her çoktan seçmeli soruyu, size veya bilginizi en iyi yansıtan tek bir seçeneği işaretleyerek cevaplayın. Sadece size en yakın seçeneği seçin.
Çoktan seçmeli soruda “Doğru olduğunu düşündüğünüz tüm seçenekleri işaretleyebilirsiniz.”, bilgisi yer alıyorsa, dilediğiniz kadar seçeneği işaretleyebilirsiniz.
Açık uçlu sorularda, soruda belirtilen sayıyı ilgili alana yazınız.

**1. Cinsiyetiniz nedir?**
A) Kadın
B) Erkek
C) Diğer

**2. Yaşınız nedir?** ________

**3. Eğitim yılınız nedir?**
A) İlk yıl
B) Orta yıllar
C) Son yıl

**4. Eğitim alanınız nedir?**
A) Tıp
B) Hemşirelik
C) Ebelik
D) Hukuk
E) Mühendislik

**5. Irkınız nedir?**
A) Beyaz
B) Siyah
C) İspanyol
D) Asya
E) Diğer

**6. Daha önce HPV’yi (Human papilloma virüsü) hiç duydunuz mu?**
A) Evet
B) Hayır

**7. HPV çok yaygın bir cinsel yolla bulaşan hastalık mıdır?**
A) Evet
B) Hayır
C) Bilmiyorum

**8. HPV enfeksiyonu her zaman klinik belirtiler gösterir mi?**
A) Evet
B) Hayır
C) Bilmiyorum

**9. Aşağıdakilerden hangisi/hangileri HPV'den kaynaklanabilir? (Doğru olduğunu düşündüğünüz tüm seçenekleri işaretleyebilirsiniz.)**
A) Hiçbir fikrim yok
B) Serviks (rahim ağzı) kanseri
C) Genital siğiller
D) Vajina kanseri
E) Vulva kanseri
F) Anal (makat) kanser
G) Penis kanseri
H) Baş ve boyun kanseri
I) HİV veya AIDS

**10. HPV nasıl bulaşır? (Doğru olduğunu düşündüğünüz tüm seçenekleri işaretleyebilirsiniz.)**
A) Hiçbir fikrim yok
B) El ele tutuşmak
C) Öpüşmek
D) Genital cilt teması
E) Halka açık tuvaletler
F) Korunmasız vajinal cinsel ilişki
G) Korunmasız oral cinsel ilişki
H) Korunmasız anal cinsel ilişki
I) Kondom kullanılan cinsel ilişki
J) Ortak kaşık ya da bardak paylaşmak
K) Hapşırma veya öksürme

**11. Daha önce HPV aşısını duydunuz mu?**
A) Evet
B) Hayır

**12. Aşağıdakilerden hangisi/hangileri HPV aşısı ile önlenebilir? (Doğru olduğunu düşündüğünüz tüm seçenekleri işaretleyebilirsiniz.)**
A) Hiçbir fikrim yok
B) Serviks (rahim ağzı) kanseri
C) Genital siğiller
D) Vajina kanseri
E) Vulva kanseri
F) Anal (makat) kanser
G) Penis kanseri
H) Baş ve boyun kanseri
I) HİV veya AIDS

**13. HPV ve HPV aşılarını duyduysanız, bunları nereden öğrendiniz? (Doğru olduğunu düşündüğünüz tüm seçenekleri işaretleyebilirsiniz.)**

A) Aile
B) Arkadaşlar
C) Sağlık çalışanları
D) Öğretmenler
E) Medya
F) Diğer

**14. HPV aşısı oldunuz mu?**

A) Evet
B) Hayır

**15. Aşıyı olduysanız (14. Soruya Evet cevabını verdiyseniz), sizi ne motive etti? (Doğru olduğunu düşündüğünüz tüm seçenekleri işaretleyebilirsiniz.)**

A) Okulda rutin aşılama
B) Serviks/diğer kanserleri önlemek
C) HPV'nin partnerime bulaşmasını önlemek
D) Siğilleri önlemek
E) HPV olan/olmuş olan birini tanıyorum
F) HPV yüzünden kanser olan/olmuş olan birini tanıyorum
G) HPV aşısı olan birini tanıyorum
I) Bilmiyorum

**16. Aşıyı olmadıysanız (14. Soruya Hayır cevabını verdiyseniz), neden olmadınız? (Doğru olduğunu düşündüğünüz tüm seçenekleri işaretleyebilirsiniz.)**

A) Bu konuyu hiç düşünmedim
B) Hakkında yeterli bilgi sahibi değilim
C) Ben istikrarlı/düzenli bir ilişki içindeyim
D) Ben bakirim/bakireyim
E) Şu anda cinsel olarak aktif değilim
F) Aşı için çok yaşlıyım
G) Pahalı olduğu için
H) Güvenilir olduğuna emin değilim
I) Aşıyı bulamadım
J) Hala kararsızım
K) İğneleri sevmem
L) Aşılara karşıyım
M) Aşı işe yaramıyor
N) Ben bir erkeğim ve kendimi risk altında hissetmiyorum
O) Zaten HPV'im var
P) Kondom kullanıyorum

**17. HPV aşısını bir çocuk veya ergen için önerir misiniz?**

A) Evet
B) Hayır
C) Bilmiyorum

**18. HPV aşısını bir arkadaş veya akrabanıza önerir misiniz?**
A) Evet
B) Hayır
C) Bilmiyorum

**19. Yaşam boyu cinsel partner sayınız nedir?**
A) 0
B) 1-5
C) 6-10
D) 10'dan fazla

**20. İlk cinsel ilişkiye girdiğiniz yaş (eğer girdiyseniz)?** ________

**21. Cinsel yöneliminiz nedir?**
A) Heteroseksüel
B) LGBTIQ

**22. Sigara kullanım öykünüz nedir?**
A) Hiç kullanmadım
B) Bıraktım
C) Sigara kullanıyorum

Anketi doldurduğunuz için teşekkür ederiz :)
