# Supplementary material for: Assessment of Knowledge and Attitudes Toward HPV and HPV Vaccine Among Students of a University in Cyprus: A Cross-Sectional Survey
Source: Vaccines (Basel). 2026 Oct 8;14(10):885. doi: 10.3390/vaccines14100885 (PMC13709563; doi:10.3390/vaccines14100885)
Supplement: Supplementary file 1 [file vaccines-14-00885-s001.zip › S4. Ethical Approval - HPV.pdf]

NEAR EAST UNIVERSITY
SCIENTIFIC RESEARCH ETHICS COMMITTEE

RESEARCH PROJECT EVALUATION REPORT

Meeting date :30.04.2025

Meeting Number :2025/133

Project number :1957

The project entitled "Evaluation of Human Papillomavirus (HPV) Infection and HPV Vaccine Knowledge, Awareness and Attitude Among University Students in Cyprus – A Cross-Sectional Study" (Project no: NEU/2025/133-1957), which will be conducted by Yrd. Doç. Dr. Burcu Özbakır has been reviewed and approved by the Near East University Scientific Research Ethical Committee.

Prof. Dr. Şanda Çalı
Near East University
Head of Scientific Research Ethics Committee

Committee Member	Role	Meeting Attendance *Attended(✓)/Not attended(X)*	Decision *Approved(✓)/Rejected(X)*
1. Prof. Dr. Şanda Çalı	Head	✓	✓
2. Assoc. Prof. Dr. Gulifeiya Abuduxike	Rapporteur	✓	✓
3. Prof. Dr. Tamer Yılmaz	Member	✓	✓
4. Prof. Dr. Şahan Saygı	Member	✓	✓
1. Prof. Dr. İlker Etikan	Member	X	X
5. Assoc. Prof. Dr. Dilek Sarpkaya Güder	Member	✓	✓
6. Prof. Dr. Burçin Şanlıdağ	Member	✓	✓
7. Prof. Dr. Nerin Bahçeciler	Member	✓	✓
